# Supplementary material for: Ketamine evoked disruption of entorhinal and hippocampal spatial maps
Source: Nat Commun. 2023 Oct 7;14:6285. doi: 10.1038/s41467-023-41750-4 (PMC10560293; doi:10.1038/s41467-023-41750-4)
Supplement: Supplementary file 1 — Supplementary Information [file 41467_2023_41750_MOESM1_ESM.pdf]

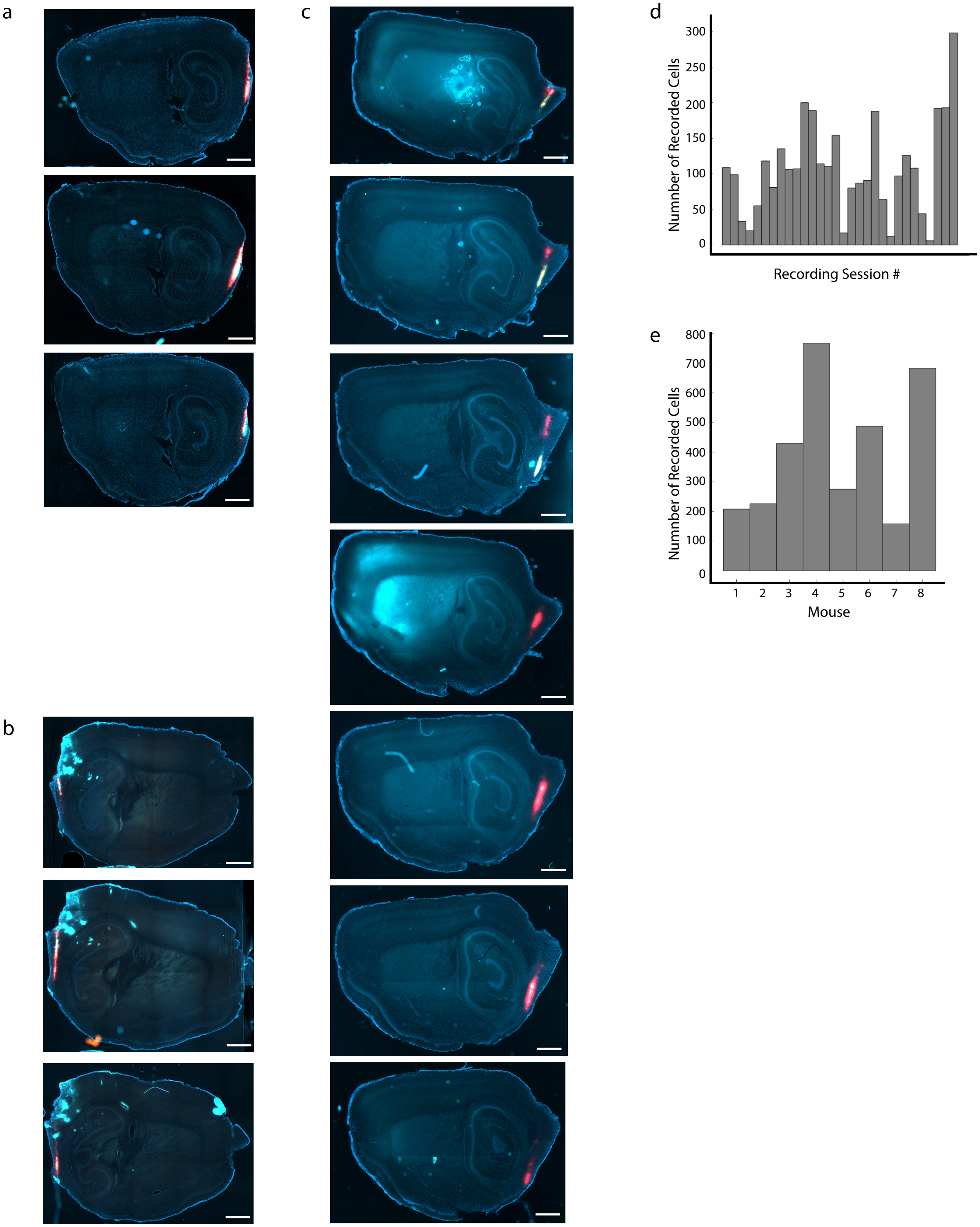
a
b
c
d
300
250
200
150
100
50
0
Numnber of Recorded Cells
Recording Session #
e
800
700
600
500
400
300
200
100
0
Numnber of Recorded Cells
1
2
3
4
5
6
7
8
Mouse

Supplementary Figure 1

a-c. Example histology of sagittal sections from three different mice (each sub panel corresponds to an individual mouse). Probe tracks are indicated by the fluorescent dye (red, yellow). Right hemisphere shown in (a), (c) and left hemisphere shown in (b). Scale bars are 1mm.

d. Number of recorded cells across all recording sessions.

e. Number of recorded cells for each individual mouse (pooled across all recording sessions).

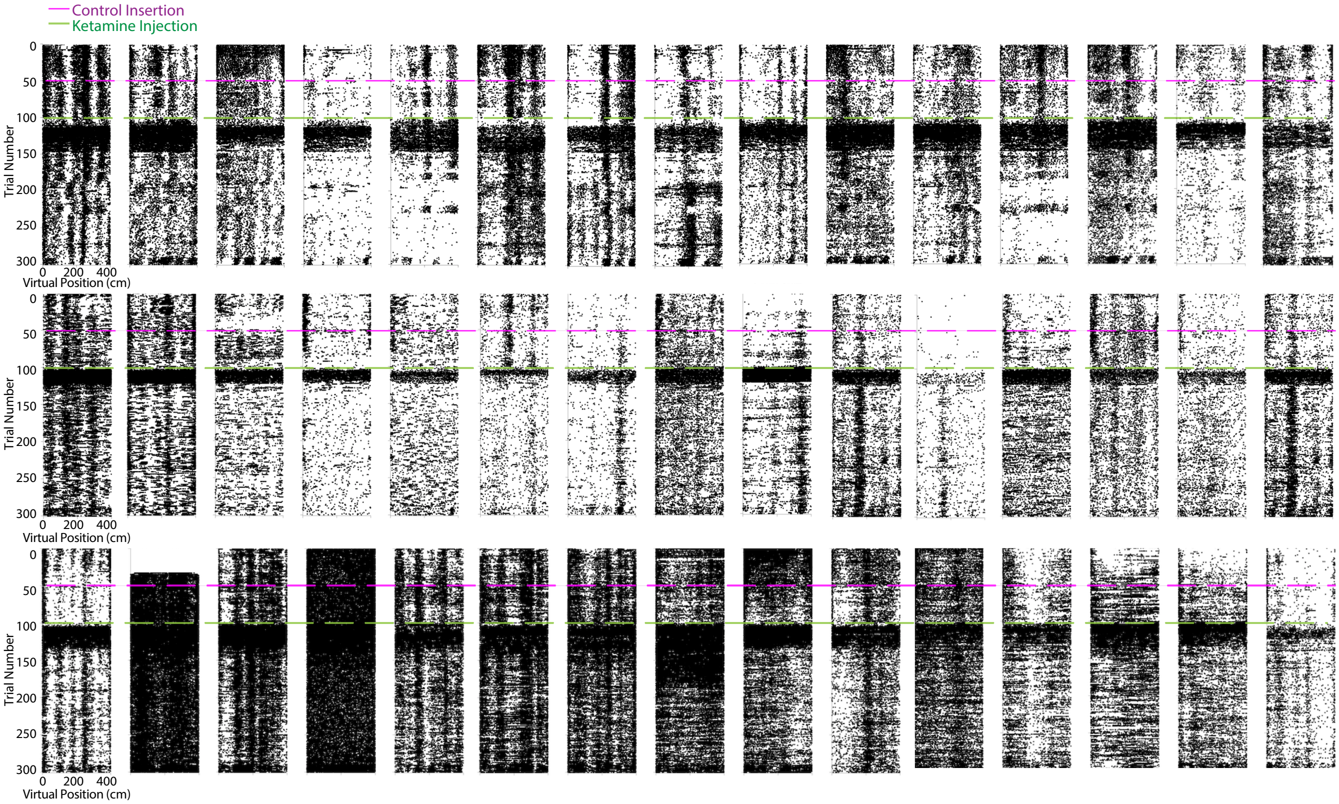

Control Insertion
Ketamine Injection
Trial Number
0
50
100
150
200
250
300
0 200 400
Virtual Position (cm)

Supplementary Figure 2

Spatial raster plots of 15 example cells from 3 different recording sessions in 3 different mice (each row is a unique session). Raster plots indicate individual spikes (black dots). The magenta line marks the beginning of the control epoch and the green line marks the beginning of the ketamine epoch.

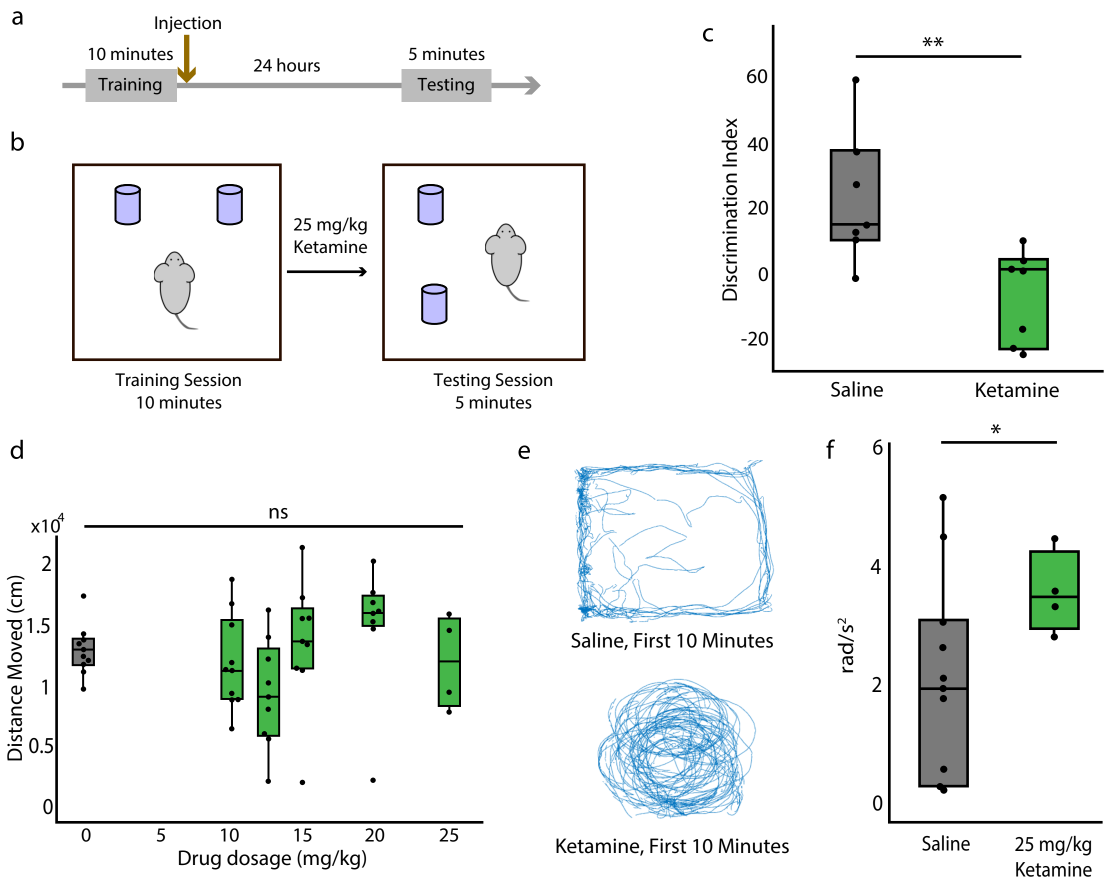

a
Injection
10 minutes
24 hours
5 minutes
Training
Testing
b
25 mg/kg
Ketamine
Training Session
10 minutes
Testing Session
5 minutes
c
**
Discrimination Index
60
40
20
0
-20
Saline
Ketamine
d
ns
x10^4
Distance Moved (cm)
2
1.5
1
0.5
0
0
5
10
15
20
25
Drug dosage (mg/kg)
e
Saline, First 10 Minutes
Ketamine, First 10 Minutes
f
*
rad/s²
6
4
2
0
Saline
25 mg/kg
Ketamine

Supplementary Figure 3

a. In the training session, mice explored two objects for 10 minutes. Immediately after the training session, half the mice were given 25 mg/kg ketamine and the other half were given a control saline injection. The following day, one of the objects was moved to a new spatial location and the mice were allowed to explore freely for 5 minutes.
b. Schematic of object-location memory tasks.
c. Discrimination index value for the 14 mice tested in the object-location memory task. Mice that received the saline injection spent significantly more time investigating the object that moved, indicating preserved spatial memory consolidation (two-sided t-test, $t(6) = 3.14$, $p = 0.008$, $n = 7$ mice per group).
d. 5 male mice and 5 female mice were given ketamine at the following doses: 0 mg/kg, 10 mg/kg, 12.5 mg/kg, 15 mg/kg, 20 mg/kg, and 25 mg/kg. The drug was delivered to mice via an IP injection and the distance they moved in a 60 cm×60 cm open field was tracked for 30 minutes afterwards. Ketamine did not have consistent dose-dependent effects on distance moved (repeated measures mixed effects model, $F(2.26, 15.85) = 1.77$, $p = 0.2$).
e. Example trajectory of an individual mouse over the 10 minutes following a saline control injection (top) and a 25 mg/kg ketamine injection (bottom). Mice that received a ketamine injection often displayed stereotyped spinning behaviors.
f. Mice that received ketamine had greater angular (Welch's two-sided t-test, $t(11) = 2.41$, $p = 0.034$, $n = 10$ control mice and 4 ketamine mice).

Significant comparisons highlighted $^*p < 0.05$, $^{**}p < 0.01$, and $^{***}p < 0.001$. For all box plots, the central mark indicates the median, the bottom and top edges of the box indicate the 25th and 75th percentiles, respectively, and the whiskers extend to the most extreme data points not considered outliers. Source data are provided as a Source Data file.

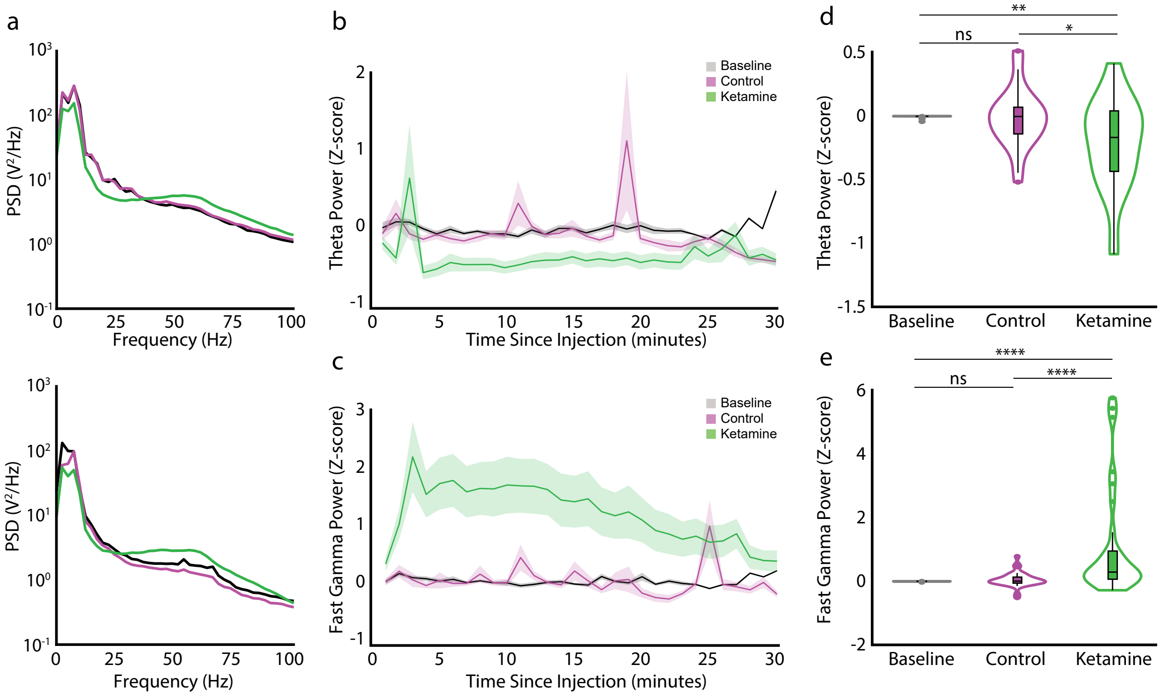

a
PSD (V²/Hz)
Frequency (Hz)
b
Theta Power (Z-score)
Time Since Injection (minutes)
Baseline
Control
Ketamine
c
Fast Gamma Power (Z-score)
Time Since Injection (minutes)
Baseline
Control
Ketamine
d
**
ns
*
Theta Power (Z-score)
Baseline
Control
Ketamine
e
****
ns
****
Fast Gamma Power (Z-score)
Baseline
Control
Ketamine

Supplementary Figure 4

a. Point source density (PSD) from two example mice during baseline trials (black), control trials (magenta), and the first 30 minutes following ketamine injection (green). A decrease in theta frequency power and increase in gamma frequency power, particularly fast gamma power, is visible.
b. Mean Z-scored theta frequency power (5-11 Hz) in each 1 minute bin 30 minutes after baseline, control injection, and ketamine injection. Power was Z-scored to the mean and standard deviation of the baseline epoch. Measured on one electrode per session (n=30 sessions)
c. Same as b, for fast gamma frequency power (50-110 Hz).
d. Z-scored theta power averaged over time in baseline trials, control trials, and the first 30 minutes following ketamine injection (n = 30 sessions). Theta power is reduced following ketamine injection (Two-sided Wilcoxon matched pairs signed rank test, vs baseline: Z = 2.73, p = 0.0064; vs control: Z = 2.22, p = 0.0262) but not following control injection (Z = 0.48, p = 0.6288). Baseline trials have a Z-score of 0 because power was normalized to the baseline epoch. To control for the effects of velocity on theta power, power in each 1 second bin during the control and ketamine epoch were sampled to match the velocities of the baseline epoch.
e. Same as d, for fast gamma frequency power. Fast gamma power increased following ketamine injection (Two-sided Wilcoxon matched pairs signed rank test, vs baseline: Z = -3.98, $p = 6.89 \times 10^{-5}$; vs control: Z = -3.88, p = 0.0001) but not following control injection (Z = -1.10, p = 0.2712).

For d and e, all violins have the same area, but the width represents the kernel probability density of the data at different values. The central mark of the boxplot indicates the median, the bottom and top edges of the box indicate the 25th and 75th percentiles, respectively, and the whiskers extend to the most extreme data points not considered outliers. Significant comparisons highlighted *p < 0.05, **p < 0.01, and ****p < 0.0001. Source data are provided as a Source Data file.

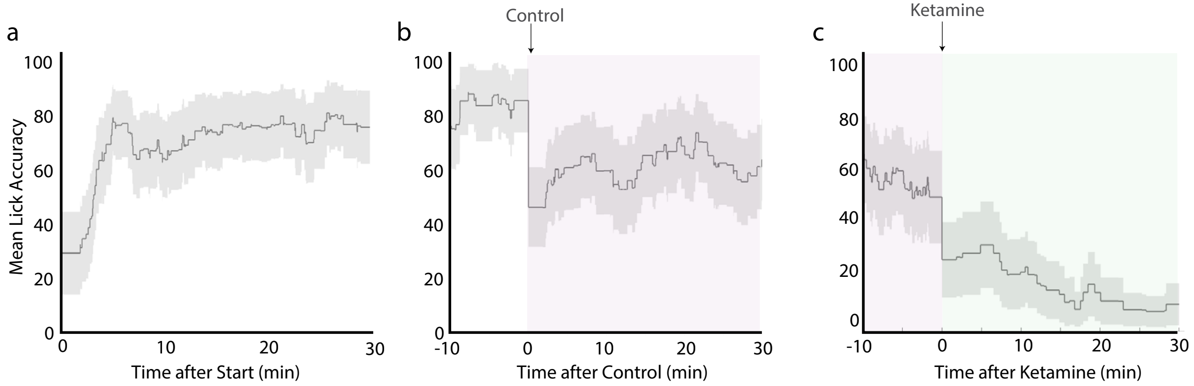
a
b
c
Control
Ketamine
Mean Lick Accuracy
0
20
40
60
80
100
-10
0
10
20
30
Time after Start (min)
Time after Control (min)
Time after Ketamine (min)

Supplementary Figure 5

a. Mean mouse lick accuracy in the first 30 minutes of the baseline epoch (n = 30 sessions, 8 mice). Solid lines represent mean lick accuracy and shaded regions represent standard error of the mean.
b. Mean lick accuracy in the 10 minutes before the control injection and the 30 minutes after the control injection (n = 30 sessions, 8 mice). Solid lines and shading as reported in (a). Control epoch highlighted in magenta. Mean lick accuracy drops in the control epoch when examining accuracy over time but then remains stable.
c. Mean lick accuracy in the 10 minutes before the ketamine injection and 30 minutes after the ketamine injection (n = 30 sessions, 8 mice). Solid lines and shading as reported in (a). Control epoch highlighted in magenta and ketamine epoch highlighted in green. Mean lick accuracy drops significantly in the ketamine epoch and approaches 0.

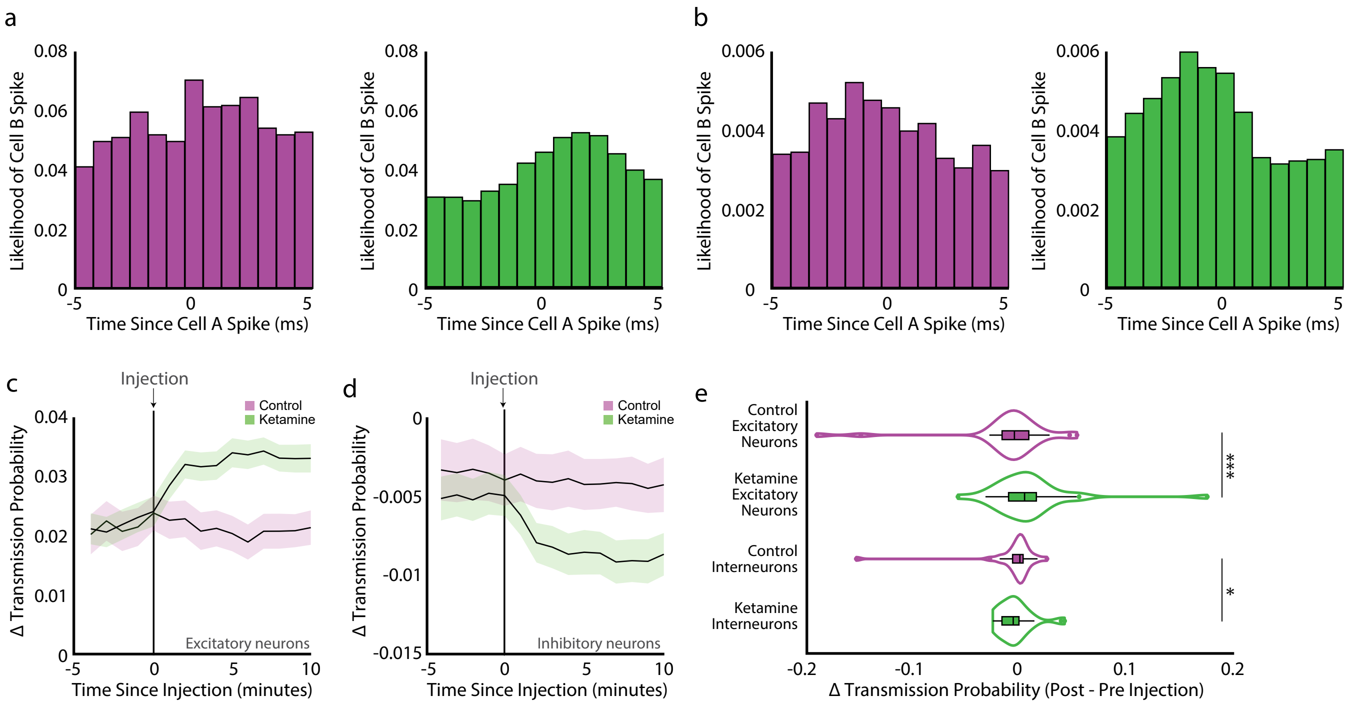

a
Likelihood of Cell B Spike
0.08
0.06
0.04
0.02
0
-5
0
5
Time Since Cell A Spike (ms)
b
0.006
0.004
0.002
c
Injection
Control
Ketamine
Δ Transmission Probability
0.04
0.03
0.02
0.01
Excitatory neurons
10
Time Since Injection (minutes)
d
-0.005
-0.01
-0.015
Inhibitory neurons
e
Control Excitatory Neurons
Ketamine Excitatory Neurons
Control Interneurons
Ketamine Interneurons
***
*
-0.2
-0.1
0.1
0.2
Δ Transmission Probability (Post - Pre Injection)

Supplementary Figure 6

a. Cross-correlogram of spiking in an example cell B relative to a spike of a putative excitatory cell A during control trials (magenta, left) and the first 30 minutes following ketamine injection (green, right). Spiking in cell B increases following a spike in cell A (0 to 5 ms vs -5 to 0 ms), indicating an excitatory connection. This increase is larger following ketamine injection.
b. Cross-correlogram of spiking in an example cell B relative to a spike of a putative inhibitory cell A during control trials (magenta, left) and the first 30 minutes following ketamine injection (green, right). Spiking in cell B decreases following a spike in cell A (0 to 5 ms vs -5 to 0 ms), indicating an inhibitory connection. This decrease is larger following ketamine injection.
c. Difference in transmission probability for putative excitatory connections in each 1 minute bin from 5 minutes prior to 10 minutes after injection (n = 5019 connections). Transmission probability is the likelihood of observing a spike from cell B in the 0.8-4.8 ms following a single spike from cell A. Difference in transmission probability is transmission probability minus the likelihood of observing a spike from cell B 0.8-4.8 ms prior to a spike from cell A, to control for increases in baseline firing rate. This difference reflects the extent to which a spike from cell A increases firing in cell B. Line indicates mean and shaded region shows SEM.
d. Same as c, for putative inhibitory connections (n = 8724 connections). This difference reflects the extent to which a spike from cell A suppresses firing in cell B.
e. Violin plot of the change in the delta transmission probability between 5 minutes before and 5 minutes after an injection of excitatory and inhibitory connections, averaged over sessions (two-sided Wilcoxon matched pairs signed rank test, excitatory: $Z = -3.38$, $p = 0.0007$; inhibitory: $Z = 2.7$, $p = 0.03$; n=30 sessions). All violins have the same area, but the width represents the kernel probability density of the data at different values. The central mark of the boxplot indicates the median, the bottom and top edges of the box indicate the 25th and 75th percentiles, respectively, and the whiskers extend to the most extreme data points not considered outliers. Significant comparisons highlighted *$p < 0.05$ and ***$p < 0.001$.

Source data are provided as a Source Data file.

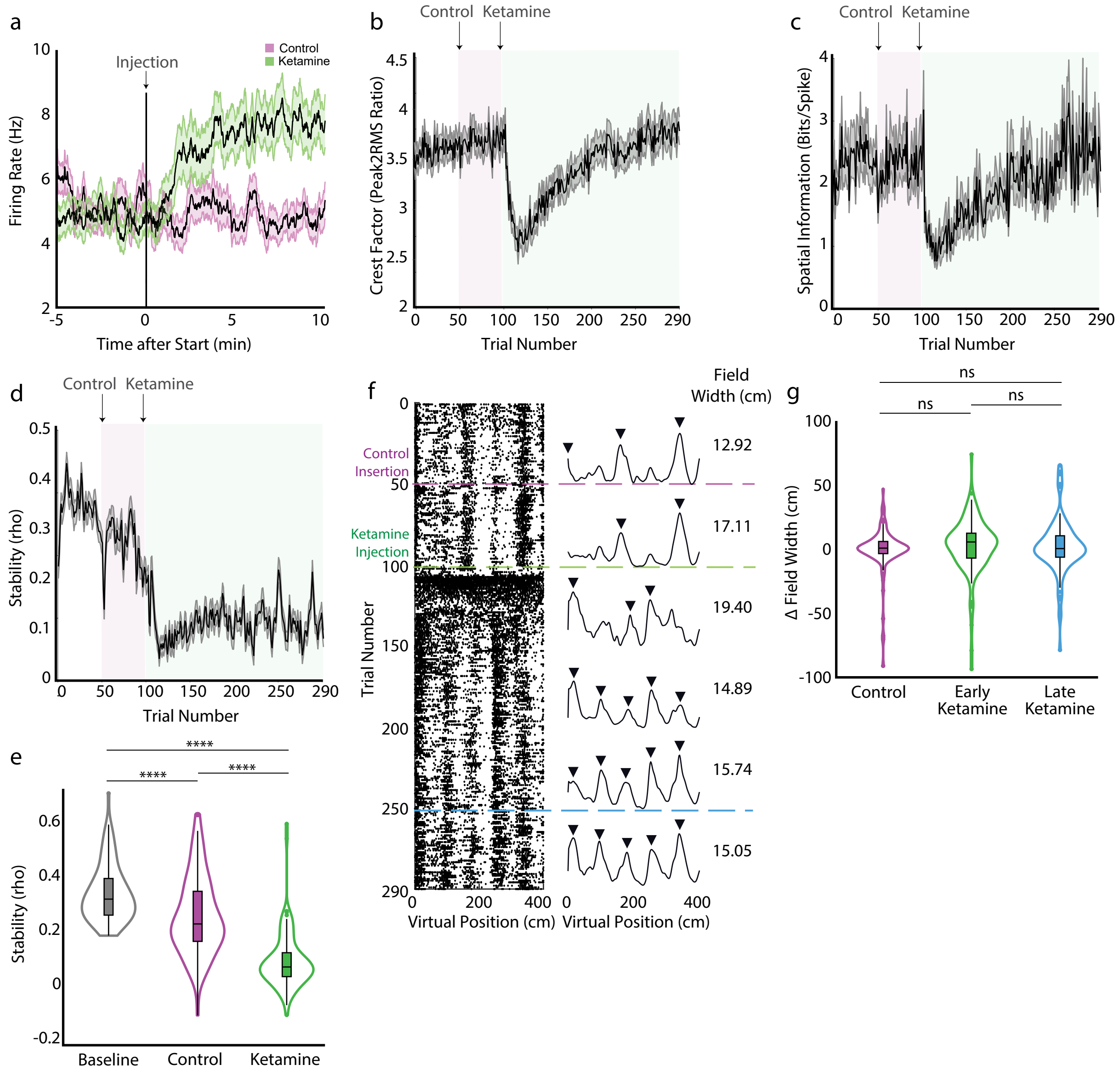
a
Injection
Control
Ketamine
Firing Rate (Hz)
Time after Start (min)
b
Control Ketamine
Crest Factor (Peak2RMS Ratio)
Trial Number
c
Control Ketamine
Spatial Information (Bits/Spike)
Trial Number
d
Control Ketamine
Stability (rho)
Trial Number
e
****
****
****
Stability (rho)
Baseline
Control
Ketamine
f
Field Width (cm)
Control Insertion
Ketamine Injection
Trial Number
12.92
17.11
19.40
14.89
15.74
15.05
Virtual Position (cm)
Virtual Position (cm)
g
ns
ns
ns
Δ Field Width (cm)
Control
Early Ketamine
Late Ketamine

Supplementary Figure 7

a. Mean firing rate of putative grid cells 5 minutes before and 10 minutes after control (magenta) or ketamine (green) injection.
b. Mean crest factor of putative grid cells (n = 102 cells, 8 mice).
c. Mean spatial information score (bits/spike) of putative grid cells (n = 102 cells, 8 mice).
d. Average spatial stability values of grid cells per trial across 300 trials (n = 102 cells, 8 mice).
e. Comparison of the mean stability of neurons in the baseline (black), control (magenta), and ketamine (green) epochs. Spatial stability decays during the control epoch ($Z = 6.31$, $p = 2.84 \times 10^{-10}$, n = 102 cells, two-sided Wilcoxon matched pairs signed rank test) and to a greater extent during the ketamine epoch ($Z = 8.6$, $p < 10^{-20}$ vs baseline epoch; $Z = 8.16$, $p = 3.33 \times 10^{-16}$ vs control epoch, n = 102 cells, two-sided Wilcoxon matched pairs signed rank test).
f. Raster plot (left) and identified spatial fields (right) of a single grid cell. Spatial fields are averaged over 50 trial blocks. Arrows indicate identified fields. Maximum field width for each map listed on the right.
g. Comparison of the change in maximum field width of grid cells between baseline epochs and control (trials 50-100, magenta, Z = 2.37, p = 0.0522), early ketamine (trials 100-150, green, Z = -1.41, p = 0.4019), and late ketamine (trials 250-290, blue, Z = 0.62, p = 0.8985) epochs. Two-sided Wilcoxon matched pairs signed rank test with Sidak correction for multiple comparisons, n = 102 cells.

For b-d, line indicates mean and shaded region shows SEM. Control epoch is highlighted in magenta (trials 51-100). Ketamine epoch is highlighted in green (trials 101-300).

For e and g, all violins have the same area, but the width represents the kernel probability density of the data at different values. The central mark of the boxplot indicates the median, the bottom and top edges of the box indicate the 25th and 75th percentiles, respectively, and the whiskers extend to the most extreme data points not considered outliers. Significant comparisons highlighted ****p < 0.0001.

Source data are provided as a Source Data file.

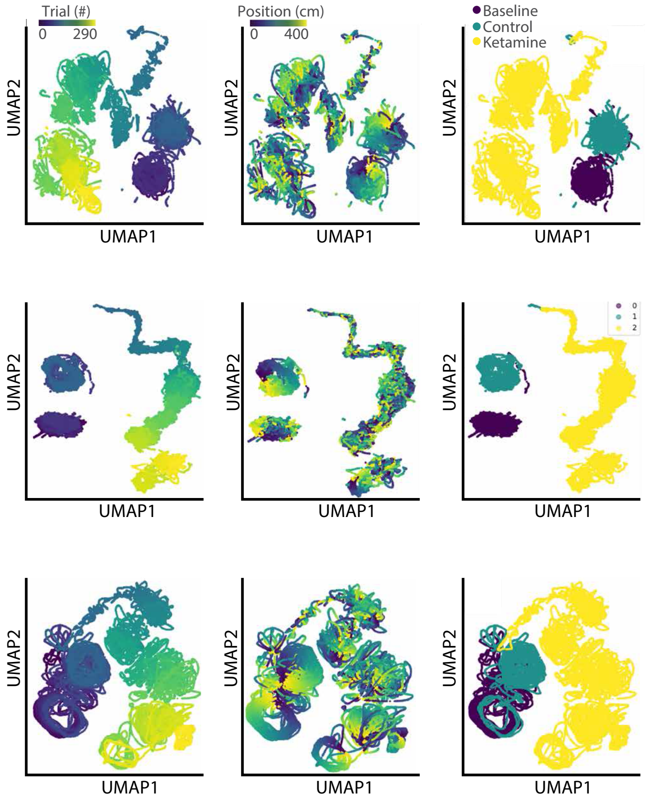
Trial (#)
0
290
Position (cm)
0
400
Baseline
Control
Ketamine
0
1
2
UMAP1
UMAP2

Supplementary Figure 8

Three examples of temporally-binned firing rates of neural populations projected onto the first two UMAP component dimensions. Colored by trial number (left column), VR position (middle column), and experimental epoch (right column).
