# Supplementary material for: Ketamine evoked disruption of entorhinal and hippocampal spatial maps
Source: Nat Commun. 2023 Oct 7;14:6285. doi: 10.1038/s41467-023-41750-4 (PMC10560293; doi:10.1038/s41467-023-41750-4)
Supplement: Supplementary file 3 — Reporting Summary [file 41467_2023_41750_MOESM3_ESM.pdf]

nature portfolio

Corresponding author(s): Lisa M Giocomo, Francis K Masuda

Last updated by author(s): Aug 9, 2023

Reporting Summary

Nature Portfolio wishes to improve the reproducibility of the work that we publish. This form provides structure for consistency and transparency in reporting. For further information on Nature Portfolio policies, see our Editorial Policies and the Editorial Policy Checklist.

Statistics

For all statistical analyses, confirm that the following items are present in the figure legend, table legend, main text, or Methods section.

n/a	Confirmed	
☐	☒	The exact sample size (*n*) for each experimental group/condition, given as a discrete number and unit of measurement
☐	☒	A statement on whether measurements were taken from distinct samples or whether the same sample was measured repeatedly
☐	☒	The statistical test(s) used AND whether they are one- or two-sided<br>*Only common tests should be described solely by name; describe more complex techniques in the Methods section.*
☐	☒	A description of all covariates tested
☐	☒	A description of any assumptions or corrections, such as tests of normality and adjustment for multiple comparisons
☐	☒	A full description of the statistical parameters including central tendency (e.g. means) or other basic estimates (e.g. regression coefficient) AND variation (e.g. standard deviation) or associated estimates of uncertainty (e.g. confidence intervals)
☐	☒	For null hypothesis testing, the test statistic (e.g. *F*, *t*, *r*) with confidence intervals, effect sizes, degrees of freedom and *P* value noted<br>*Give P values as exact values whenever suitable.*
☒	☐	For Bayesian analysis, information on the choice of priors and Markov chain Monte Carlo settings
☒	☐	For hierarchical and complex designs, identification of the appropriate level for tests and full reporting of outcomes
☐	☒	Estimates of effect sizes (e.g. Cohen's *d*, Pearson's *r*), indicating how they were calculated

Our web collection on statistics for biologists contains articles on many of the points above.

Software and code

Policy information about availability of computer code

Data collection	Electrophysiological data were collected using Phase 3B Neuropixels 1.0 silicon probes, digitized with a CMOS amplifier and multiplexer built into the electrode array, and then written to disk using SpikeGLX software version 20190327-phase3B2. Ca+ imaging data were acquired using custom-developed and validated software (Compiled DAQ Software OLD, https://github.com/daharoni/Miniscope_DAQ_Software version 1). Open field and object location memory behavior were acquired by EthoVision XT version 14.
Data analysis	Analyses were carried out using MATLAB and Python scripts. MATLAB was used to perform statistical calculations. Cell clusters were isolated using Kilosort 2.0 and manually reviewed using Phy. All analysis code are available at https://github.com/GiocomoLab/Masuda_et_al_2023.

For manuscripts utilizing custom algorithms or software that are central to the research but not yet described in published literature, software must be made available to editors and reviewers. We strongly encourage code deposition in a community repository (e.g. GitHub). See the Nature Portfolio guidelines for submitting code & software for further information.

Data

Policy information about availability of data

All manuscripts must include a data availability statement. This statement should provide the following information, where applicable:

- Accession codes, unique identifiers, or web links for publicly available datasets
- A description of any restrictions on data availability
- For clinical datasets or third party data, please ensure that the statement adheres to our policy

The MEC binned firing rates, hippocampal calcium traces, and mouse position data generated in this study have been deposited in the Figshare database under accession code doi:10.6084/m9.figshare.22696309 [https://figshare.com/articles/dataset/Ketamine_evoked_disruption_of_entorhinal_and_hippocampal_spatial_maps/22696309]. Source data are provided with this paper.

Human research participants

Policy information about studies involving human research participants and Sex and Gender in Research.

Reporting on sex and gender	NA
Population characteristics	NA
Recruitment	NA
Ethics oversight	NA

Note that full information on the approval of the study protocol must also be provided in the manuscript.

Field-specific reporting

Please select the one below that is the best fit for your research. If you are not sure, read the appropriate sections before making your selection.

☒ Life sciences ☐ Behavioural & social sciences ☐ Ecological, evolutionary & environmental sciences

For a reference copy of the document with all sections, see nature.com/documents/nr-reporting-summary-flat.pdf

Life sciences study design

All studies must disclose on these points even when the disclosure is negative.

Sample size	Sample sizes were based upon on convention in the field. The sample sizes were not calculated by power analyses, however, our sample sizes are comparable or higher than those previously used in the literature (Low et al, 2021 and Campbell, Attinger, et al, 2021).
Data exclusions	Cells with low firing rates (< 100 spikes) and recording sessions with <10 cells were excluded. Mice were deemed fully trained on the virtual reality track and ready to record when they completed 400 trials within 1 hour for two consecutive days. Mice that never learned the task were excluded from further experiments.
Replication	Up to 14 mice were used as a cohort for each batch of experiments. Key experiments were repeated with at least two different cohorts of mice and the results are reproducible.
Randomization	Animals were randomly assigned to experimental groups.
Blinding	Experimenters were not blinded to experimental groups. Experimental groups received drug treatments which produced obvious behavioral effects, thus by witnessing the animal undergoing the experiment, any blinding that would have been attempted would be compromised. It would be impossible to blind the experimenter to the experimental conditions while still being physically present during the experiment. Presence during the experiment is required by the ethics committee overseeing the experiment.

Reporting for specific materials, systems and methods

We require information from authors about some types of materials, experimental systems and methods used in many studies. Here, indicate whether each material, system or method listed is relevant to your study. If you are not sure if a list item applies to your research, read the appropriate section before selecting a response.

Materials & experimental systems

n/a	Involved in the study
☒	☐ Antibodies
☒	☐ Eukaryotic cell lines
☒	☐ Palaeontology and archaeology
☐	☒ Animals and other organisms
☒	☐ Clinical data
☒	☐ Dual use research of concern

Methods

n/a	Involved in the study
☒	☐ ChIP-seq
☒	☐ Flow cytometry
☒	☐ MRI-based neuroimaging

Animals and other research organisms

Policy information about studies involving animals; ARRIVE guidelines recommended for reporting animal research, and Sex and Gender in Research

Laboratory animals	Mice were C57BL/6 and 129S6/SvEvTac and aged 12 weeks to 24 weeks. A194; CaMKIIa-tTA; CaMKIIa-Cre triple transgenic mice were used (Jackson Laboratory stock #024115, 8 -12 weeks old) for the calcium imaging experiments. Animals were housed at 21–23°C and 30–70% humidity.
Wild animals	No wild animals were used in this study.
Reporting on sex	Male and female mice were used as data subjects for the electrophysiology experiments, calcium imaging experiments, and open-field experiments.
Field-collected samples	No field-collected samples were used in this study.
Ethics oversight	All procedures were approved by the Institutional Animal Care and Use Committee at Stanford University School of Medicine.

Note that full information on the approval of the study protocol must also be provided in the manuscript.
